# Perceptions and Emotional State of Mothers of Children with and without Microcephaly after the Zika Virus Epidemic in Rural Caribbean Colombia

**DOI:** 10.3390/bs10100147

**Published:** 2020-09-25

**Authors:** Kelly Romero-Acosta, Elena Marbán-Castro, Katy Arroyo-Alvis, Germán Arrieta, Salim Mattar

**Affiliations:** 1 Faculty of Humanities and Education, Corporación Universitaria del Caribe CECAR, Sincelejo 700001, Colombia; kelly.romero@cecar.edu.co (K.R.-A.); katy.arroyoa@cecar.edu.co (K.A.-A.); GERMAN.ARRIETA@cecar.edu.co (G.A.); 2 Department of Maternal, Child, and Reproductive health, ISGlobal, Hospital Clínic-Universitat de Barcelona, 08036 Barcelona, Spain; 3 Clinica Salud Social SAS, Sincelejo 700002, Colombia; 4 Instituto de Investigaciones Biológicas del Trópico, Universidad de Córdoba, Montería 230002, Colombia; smattar@correo.unicordoba.edu.co

**Keywords:** zika, women, microcephaly, perceptions, emotional state, grounded theory

## Abstract

Zika virus (ZIKV) infection during pregnancy can cause neurological manifestations such as microcephaly. The aim of this study was to explore perceptions of ZIKV and mental health in women exposed to ZIKV during pregnancy in Colombia. This was a mixed-methods study based on structured interviews and psychological tests. Structured interviews were transcribed and analysed with Atlas Ti software. A grounded theory approach was applied. Quantitative analysis was performed with Statistical Package for Social Science, SPSS, V. 20. The study was approved by the Ethics Committee of the Universidad de Córdoba, Montería. Seventeen women participated in the study; nine of them were mothers of children with microcephaly. Maternal age ranged from 16 to 41 years old. The main themes discussed during interviews were: feelings, support, sources of information, and consequences on children’s health. Women with children affected by microcephaly showed worse mental health compared to women with normocephalic children. Maternal mental health worsened after 24 months from giving birth. Perceptions regarding disease severity and lack of knowledge were considered to affect maternal mental health. Social support and spirituality were key determinants for caregivers. Future research is needed to further study coping mechanisms and mental health outcomes over time by affected populations.

## 1. Introduction

Zika virus (ZIKV) is an arbovirus transmitted by Aedes spp. mosquitoes that usually causes mild disease with flu-like symptoms in immunocompetent adults [1]. The re-emergence of ZIKV in 2015 in Brazil spread to almost all countries in the American region, and its neurological manifestations in fetuses led the World Health Organization (WHO) to declare the ZIKV epidemics a Public Health Emergency of International Concern [2]. ZIKV infection during pregnancy is a cause of poor pregnancy outcomes such as spontaneous abortions, microcephaly, and other severe congenital anomalies [3,4,5]. Neonates born with congenital Zika syndrome (CZS) present severe microcephaly, eye loss, brain damage (including ventriculomegaly, intracranial calcifications, and skull collapse), congenital contractures, and hypertonia [6,7]. Healthy at birth not-infected children with in-uterus exposure to ZIKV may also develop psychomotor delays, poor reactions to external stimulation, generalized spastic dystonia, and other neurodevelopmental delays during childhood [8,9,10].

Children with CZS require special health support to reach their full potential [11]. The greater part of the affected populations lives in constraint-resource settings where making the most out of available resources is a major challenge [11]. Caregivers of children with disabilities are a group particularly susceptible to developing poor mental health, including depression, anxiety, and stress [12,13]. Furthermore, they might suffer an intensification of emotional symptoms, and also unhealthier habits, due to delays in children’s neurodevelopment over the years [14]. Usually, main caregivers of these children are their biological mothers. Maternal connection depends on the ability to adequately understand and respond to children’s needs [15,16], and it could be affected by maternal anxiety and depression [17].

Previous studies have reported significant associations between maternal psychological distress in caregivers of children with disabilities compared to families with healthy children [14,18,19,20]. Research stresses that healthcare professionals need to pay special attention to those parents’ mental health and integrate coping strategies and the right social support to reduce stress and other burdens experienced by these families [21,22,23]. These strategies are extremely important for families living in low-resource settings, with low social support and income. Moreover, maternal mental health repercussions due to children’s disabilities caused by infectious diseases during pregnancy have not been studied in depth.

Only a few studies have explored mental health outcomes and the emotional state of women exposed to ZIKV in families carrying ZIKV-associated microcephalic children [13,24,25,26,27,28], all of them were performed in Brazil. There was one cross-sectional study where stress, anxiety, and depression were reported from 163 caregivers of children with microcephaly vs. 324 caregivers of healthy children [13]; another cross-sectional survey study with 50 caregivers of affected children without a comparison group [28]; and only one qualitative study based on participant observations [26]. There is also an Internet-based computer-mediated communications analysis study to understand experiences and perceptions of families with microcephalic children by videos published in Youtube [27]; one letter [24] and one commentary [25] to call for attention on this topic.

A previous study from our research group described the neurological abilities among children with and without microcephaly in Sucre, Colombia [29], and the present study focuses on their mothers. These families represent an extremely vulnerable group, as they belong to a low-resource setting and these women experienced the first Zika virus epidemic with consequences on reproductive health while pregnant in 2016. For that reason, we assume they might suffer emotional anxiety, depression, and general perception of poor health. However, we are also interested in further exploring qualitative data obtained from study interviews with this specific population group in order to broaden the scarce literature of the emotional state of mothers exposed to ZIKV during pregnancy in Colombia. We aim to further understand perceptions of Zika virus and mental health among mothers infected during pregnancy and to compare the emotional state of women carrying microcephalic versus normocephalic children in Sucre, Colombia. 

## 2. Materials and Methods 

### 2.1. Study Design

**Figure 1:**
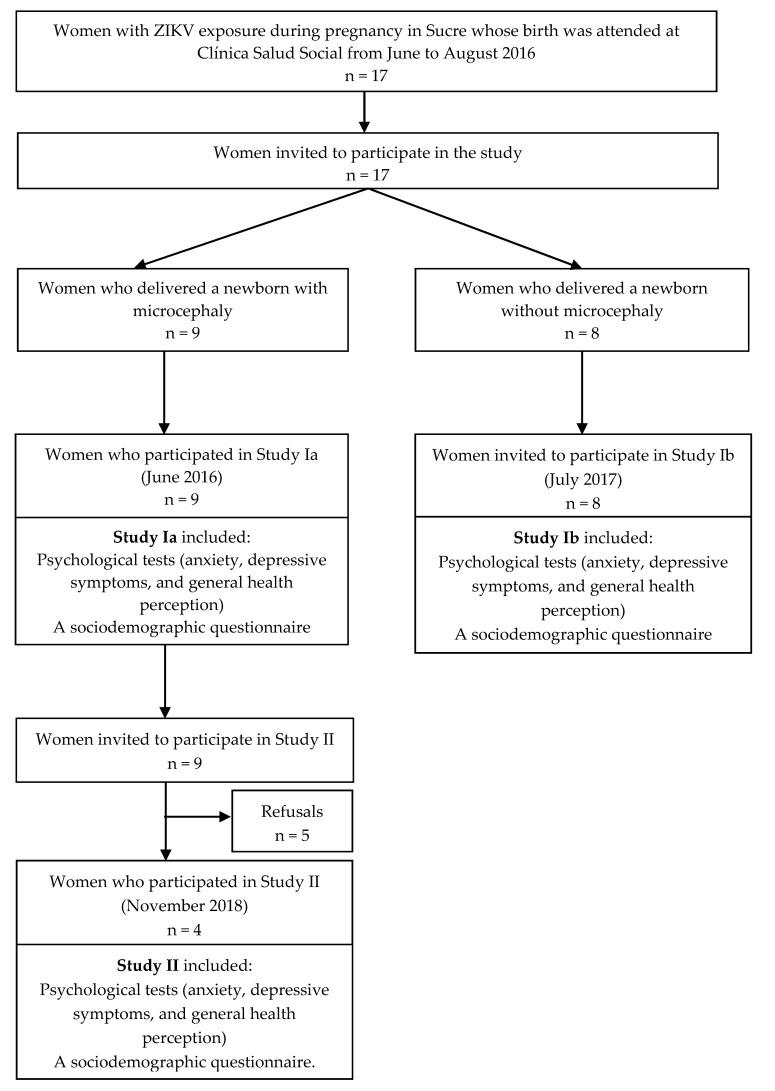
Patient flow chart of maternal enrolment in Zika virus study in Sucre, Colombia in 2016 and 2017.

This was a mixed-methods study based on structured interviews and psychological tests performed on women exposed to ZIKV during pregnancy in 2015 and 2016. Study design took place in three different phases according to the population’s sub-groups and timeline. In 2016, study Ia was developed to conduct psychological tests (anxiety, depressive symptoms, and general health perception) and a sociodemographic questionnaire to those women who delivered a microcephalic infant. In 2017, the same tests were conducted with mothers of normocephalic infants, and a structured interview was performed (Study Ib). These women were not further contacted. In 2018, women who participated in Study Ia were contacted for performance of the psychological tests and the structured interview (Study II). Figure 1 describes the study and participants flow chart.

### 2.2. Study Participants

Study participants were women enrolled in a surveillance ZIKV study conducted at a secondary health center (Clínica Salud Social) in Sincelejo, Sucre District in Colombia. Pregnant women with compatible symptoms for ZIKV infection and pregnant women with fetal anomalies detected by prenatal ultrasound were invited to participate in the study. Written informed consent was obtained prior to enrolment.

Pregnant women with possible ZIKV exposure during pregnancy in 2015 and 2016 were contacted by phone calls and invited to attend the study clinic.

### 2.3. Methods Used

The qualitative approach included a structured interview performed by a senior investigator [30]. The interview guide included questions such as “Who do you live with? Who supports you in housekeeping and child-caring? Do you have time for yourself? How do you feel regarding maternity? Did you have psychological support?” Interviews were performed in Spanish. Translation into English was carried out at the time of the analysis.

The quantitative assessment was performed with two groups of women: (a) women with suspected ZIKV infection during pregnancy carrying microcephalic children, and (b) women with confirmed ZIKV infection during pregnancy carrying normocephalic children. The first group of women attended two visits (June 2016, and November 2018). The second group was only contacted to attend on June 2017. All women were evaluated on the following psychological screening tests: (1) The State-Trait Anxiety Inventory (STAI) [31]; (2) The Self-Rating Depression Scale [32,33,34]; and (3) The General Health Questionnaire [35] A sociodemographic questionnaire using a Hollingshead index based on parental education, occupation etc., was also administered to participants [36].

### 2.4. Analysis

Structured interviews were transcribed and entered into qualitative software Atlas Ti. The analysis was performed to classify transcripts into codes and subcategories within them, applying a grounded theory approach. We analyzed quantitative data with statistical software Statistical Package for Social Science, SPSS, V. 20 (IBM, Chicago, USA). Sociodemographic data were categorized with frequencies and percentages. Scores were analyzed and presented with their standard deviations (SD). Microcephaly was defined as a head measurement at birth below 2 SD for age and sex [37].

### 2.5. Ethical Issues

The study was approved by the Ethics Committee of the Universidad de Córdoba, Montería, on 15 January 2016, under the number FMVZ-001-2016. Ethics committee reference numbers approved the study F-GI-IV-001, 10 October 2015, Clinic Salud Social, Sincelejo, Colombia, and Instituto de Investigaciones Biológicas del Trópico, University of Cordoba, Colombia # 003, 10 March 2016. We strictly followed the ethics protocols of the Minister of Health of Colombia and the Helsinki statement. Patient confidentiality was kept during and after the study.

### 2.6. Baseline Characteristics of Study Participants

Seventeen women exposed to ZIKV during pregnancy, who gave birth in 2016, participated in the study. Nine of them were carrying children with microcephaly. Children with microcephaly presented alterations such as intracranial calcifications, ventriculomegaly, and severe microcephaly, among others [29]. Maternal age ranged from 16 to 41 years old (mean of 24 years old, SD 6.52). All of them belonged to the lowest socioeconomic status. Eight women had normocephalic children, and their ages ranged from 16 to 35 years old (mean of 29 years old, SD 8.28). Two of them belonged to a lower-middle socioeconomic status (25%) and six of them belonged to the lowest socioeconomic status (75%). 

All of them lived in semi-urban areas. All of them presented clinical symptoms compatible with ZIKV infection during pregnancy and delivered at Clínica Salud Social, Sincelejo (Sucre), Colombia. Participants’ flow chart is detailed in Figure 1.

## 3. Results

### 3.1. Maternal Health of Mothers with Normocephalic vs. Microcephalic Children

Mothers of normocephalic children showed better self-perception of health, with statistically significant results, and mental health outcomes (anxiety and depression) compared to mothers of children with microcephaly, though without significant results. See Table 1.

The emotional state of mothers with children with microcephaly worsened over time. Maternal anxiety levels were higher in the second assessment, during the second year of their child’s life. Self-perception of health worsened compared to first-year evaluation. Depression remained with the same mean in both time-points. See Table 2.

### 3.2. Perceptions of Women Exposed to ZIKV during Pregnancy 

A structured interview was performed with four women carrying microcephalic children, and eight women with normocephalic children. In the second group, four topics arose.

Among all the interviews with women who were exposed to ZIKV during pregnancy (*n* = 12), common topics came up, such as feelings and support. Two additional topics were discussed during some interviews, including sources of information and consequences on children’s health. Table 3 and Table 4 resume categories, codes, and quotes from mothers with normocephalic and microcephalic children.

### 3.3. Feelings

During study interviews, mothers expressed their concerns about Zika, and directly mentioned or indirectly expressed some emotions felt during the process of the pregnancy and childcare. All the interviewed mothers expressed having experienced fear during gestation thinking about possible consequences on their children health. The emotions that were most mentioned were: distress, worry, sadness, blame, loneliness, and negative perceptions of situations. Attitudes generated by experiencing these feelings led to actions such as crying and isolation. Stress and anxiety generated thoughts of not being capable of managing the situation (sick child, caring for other family members, exclusion from relatives, etc.) and led mothers to blame themselves: “*Sometimes, stress is so intense that I forgot that my other child is six years old, and she needs my affection and love*.” Feelings experienced during pregnancy were common in both groups of women; differences were seen once the baby was born.

### 3.4. Support

The news of a positive or suspected ZIKV diagnosis during pregnancy was a time of stress and emotional distress for the studied population. Women trusted in their partner’s and relatives’ support for overcoming this situation. Interestingly, spirituality was raised up as a coping mechanism in the group of women with normocephalic children, although this question was not directly asked in the four interviews with mothers of microcephalic children. Lack of support was mentioned in this sub-group of women with affected children. Those comments of lack of support were referred to their current situation, once the baby was born, when they reported less partner support and sometimes, stigmatization of their child by the community.

### 3.5. Sources of Information

The most common sources of information for the study population were: mass media (including Internet, TV, and radio), people they trusted (from the community—such as neighbors, religion groups—and healthcare professionals). “*Well, the first time I heard it from a friend and told me. Because in Brazil… because at that time we did not know, so she told me that some children were born with small heads. She did not tell me the name, just that they were born with small heads due to Zika, a lot of children were born like that. But I told her that we had to wait, he had to wait in the name of Jesus Christ*.” Lack of knowledge from healthcare professionals could lead to misunderstandings and misinform the mothers, resulting in a worse perception of their child’s health: “*They told me [healthcare professionals] that my baby could be born with problems, that he could be born with Down syndrome*.”

### 3.6. Consequences on Children’s Health

Perceptions and concerns about children’s health were derived in a great number of occasions from popular misbeliefs and stigmatization of the disease: “*And with Zika… imagine! Going with a deformed baby, that many people said that they were like little monsters. They told me that they were little monsters because they were born just with part of their face.”* Lack of solid knowledge about consequences on children’s health led to greater perceptions of severity and mental health symptoms, being translated again in feelings such as fear, worry, etc.

## 4. Discussion

To our knowledge, this is the first study reporting perceptions regarding ZIKV infection and maternal mental health outcomes (anxiety, depression, and self-perception of health) in women exposed to the virus during pregnancy carrying normocephalic or microcephalic children, in Colombia. Though research in mental health of parents with disabilities is vast, this is not the case for families with children with ZIKV-associated microcephaly. Prior research found that vector-borne viruses cause not only clinical malaise but also emotional symptoms; as an example, 50% of individuals diagnosed with Chikungunya perceived that it affected their emotional state [16]. In line with other studies, we found that feelings of pain, sadness, and hopelessness, guided by uncertainties about the child’s health and lack of healthcare professionals’ clarity, lead to poor mental health outcomes, anxiety, worry, depression, and stress [27]. During pregnancy, negative perceptions about ZIKV and possible consequences on children’s health were similar for both groups of women, and long-term implications of maternal health outcomes were present in families with microcephalic infants [25].

Physical therapy, adequate stimulation, and control of seizures are the only treatments for these children, and those interventions highly depend on caregivers’ economy, time, educational level, and health. The educational level, including having the right and updated ZIKV information, is decisive to respond to women and children’s needs. Lack of reliable information and uncertainties enhanced feelings such as worry and blame that could lead to worsening mental health outcomes. Quality of information received by health professionals, family members, and media, among others, may influence women’s knowledge, behavior, and emotional state. An online survey of pregnant women in the USA found that 71.1% of pregnant women read about ZIKV for the first time on the Internet [38]. Contrary to that, in our population, health care professionals were the main trusted source of information for patients affected by ZIKV to have reliable information about the infection. It is highly recommended that health staff receive appropriate training material and updated protocols and guidelines to give clear messages to the population, to avoid misunderstandings, and wrong messages, such as confusing Down syndrome with Congenital Zika syndrome. Lack of updated information by healthcare professionals affects reliability in health systems. Governments and health educators should provide reliable information when informing about infectious diseases and their risks, mainly for emerging epidemics [39].

Most women in our study reported social and familiar support as a critical element in this process. Partners and relatives’ support, mainly grandmothers, were crucial during pregnancy follow up. Many participants considered their faith in God as the most important coping mechanism. Religiosity and spirituality are associated with stress coping in situations that imply death or chronic disease in children [40,41]. In our population, only mothers of healthy children were asked about religious beliefs; thus, it may seem they were the only ones reporting spirituality as a coping strategy. A further research study with in-depth interviews in the sub-group of women with microcephalic children addresses this one and other questions [data still not published].

The assessment of mental health of mothers with normocephalic children was overall good. These results were expected because uncertainties and feelings of worrisome were detected during pregnancy, but when mothers delivered a healthy baby, their emotional state improved as feelings of worrisome decreased. In the long term, this low perception of risk could be a problem leading to lower attendance to medical visits and under-detection of neurodevelopmental disabilities, because healthy-at-birth infants born to mothers with confirmed ZIKV infection still need long-term follow up to detect neurodevelopmental delays [7,12].

Maternal mental health still constitutes a problem in this special and vulnerable population. A long-term follow-up plan to monitor the emotional state of mothers with microcephalic children is needed in order to maintain mental health and improve both mothers’ and children’s health. Government support is needed in order to respond to ZIKV affected families’ needs during children’s lifespan. Research on ZIKV affected populations needs to be translated into better health for children and a better quality of life for their families. National and local strategies are thus required to improve (1) information about Zika, (2) counseling for maternal mental health, and (3) care for affected children.

There are several strengths and limitations to be considered. This study included not only quantitative but qualitative data to address a broad knowledge gap regarding caregivers of children with ZIKV-associated microcephaly; but it also addressed the importance of mental health during the whole pregnancy, as we also included mothers of normocephalic children who had a positive ZIKV diagnosis. In terms of limitations, mental health is a broad topic and we only evaluated three areas with one test each. Second, the sample size was small, hampering statistical tests from being performed to derive causal inferences among groups. There could be several variables influencing maternal mental health, such as being the first child of the family, already having affected children, maternal occupation, or capacity to maintain other tasks and activities apart from caregiving. As we did not perform a multivariable quantitative analysis, we did not take into consideration those variables. Another limitation was that for the second study visit, only mothers with microcephalic children were invited to attend the study clinic. Thus, the mental health of mothers with normocephalic children could not be assessed in the second year. Results from this study could not be generalized, as participants were very homogeneous and might differ from results performed with populations in other settings (higher socioeconomic status, population living in rural areas, well-educated women, other caregivers not only biological mothers, among others). Nonetheless, the majority of women affected by the 2015 ZIKV epidemics in Latin American countries belonged to low resource settings and the lowest socioeconomic status, such as the women in our study. In this study, we focused on biological mothers, because they are the principal caregivers of these children, but future research could be directed to study repercussions on other caregivers, such as fathers, grandparents, and also siblings of affected children, including minors and adolescents.

## 5. Conclusions

Women exposed to ZIKV during pregnancy were at risk of suffering poor mental health, such as a worse self-perception of health, depression, and anxiety. Mental health tended to worsen over time in mothers carrying children with microcephaly. Perceptions regarding ZIKV severity and lack of reliable sources of information were considered to be affecting maternal mental health. Social support, including partner’s and relatives’ support, and spirituality, were key determinants of caregivers’ coping mechanisms. Future research needs to further study coping mechanisms, maternal mental health outcomes over time, barriers, and facilitators to access healthcare services by most affected populations by the Zika epidemics.

## Figures and Tables

**Table 1:** Comparison of the emotional state of mothers carrying microcephalic (cases, *n* = 9) and normocephalic (control, *n* = 8) children.

Health Area	Category	Average	SD	*p*-Value
Self-perception of general health	Case	22.44	4.003	0.000
	Control	41.37	4.565	
Depression	Case	20.89	7.322	0.392
	Control	17.62	7.945	
Anxiety	Case	20.11	7.557	0.146
	Control	13.75	9.528	

In the scale of self-perception of general health low numbers are indicative of lower perception of health, whereas in the scales for Depression and Anxiety high numbers are indicative of worse mental health outcomes.

**Table 2:** The emotional state of mothers carrying microcephalic children in two-time points.

Category	Time Points	Minimum	Maximum	Average	SD
Self-perception of health	Year 1	33	44	38.75	4.573
	Year 2	15	27	22.75	5.315
Depression	Year 1	12	29	17.50	7.767
	Year 2	11	32	17.50	9.815
Anxiety	Year 1	14	25	19.25	4.573
	Year 2	12	31	22.50	7.853

Categories “minimum” and “maximum” correspond to the minimum and maximum scores in tool used.

**Table 3:** The emotional state of mothers carrying microcephalic children.

Category	
Code	Quote
Feelings	
Loneliness	*“I feel alone, and sometimes it makes me make a mess [the house]”*
Blame	*“Sometimes, stress is so intense, that I forgot that my other child is six years old, and she needs my affection and love”* *“He [husband] tells off the girl and demands her more than what he should”*
Patience	*“It’s a bit stressful to know that development goes slow and that you previously had a normal child who walked when he was a year of age. But I am patient, and I know that this baby’s achievements are slow, and we applaud his progress”*
Overwhelmed	*“I got stressed with medical appointments and with doctors”* *“I feel a bit overwhelmed for the child, I go with her to psychical therapy, occupational therapy, speech therapist…”*
Sadness	*“I take a towel, and scream, and cry alone”*
Gratitude	*“I feel happy. My daughter is a strong reason to be in charge. Even, she is not what we were expecting… we are satisfied now for what we have achieved”*
Support	
Lack of support	*“I do not count on my mother-in-law, neither my sister-in-law, I think they step away from me because of the baby [and starts crying]”* *“My husband works a lot, I do not count on him”*
Partner support	*“My partner was fundamental. He told me: ‘He [baby] is normal, do not worry’ ”*
Relatives support	*“Sometimes I have time for myself. Sometimes I go out to have my hair or nails done. I count on my mother’s support.”*

**Table 4:** The emotional state of mothers carrying normocephalic children.

Category	
Code	Quote
Feelings	
Sadness	*“I was feeling very bad, very sad”*
Overwhelmed	*“I felt really stressed”*
Sadness	*“I did not even want to eat. I did not want anybody to tell me anything, because I was tired of this…”*
Fear	*“I was scared”*
Gratitude	*“I would be happy anyway, to have my baby girl, because it was my desire, to have my baby. Daddy was so happy; he was going to have his first baby girl”*
Support	
Spirituality	*“I went to church and they prayed for me. They told me ‘Your baby is going to be born healthy, your baby is strong, your baby is not going to have any illness’”* *“I was sad, but at the same time, I held on God”* *“I only held on my God, that I did not have Zika”* *“They told me ‘Well, girl, just be faithful to God, that this didn’t happen to you, just that, there are people hospitalized’ and I wasn’t”*
Partner and relatives support	*“I thought of my baby, and when my husband found it out, he told me ‘You are not going to work’. And was looking after me at home. My mother-in-law helped me at home because I had Zika”*
Family support	*“They told me to come here [to the Clinic], to tell you [doctors] that I had Zika in order to have more exams performed, my neighbor told me.”*
Sources of information	
By mass media	*“Before pregnancy, you watched on TV that children could come with problems, that the baby comes with problems… What can I tell you? I don’t know the word, I forgot it… Well, more or less I had some idea about Zika. Because I searched […] on the internet to see how one was ill, to see how you feel [with Zika] more or less… The day I had the rash, I searched on the internet to see if it was it [Zika]. But there they said that you had joint pain, but I didn’t have it, just some mosquito bites, but nothing else…”*
By healthcare professionals	*“They told me [healthcare professionals] that my baby could be born with problems, that he could be born with Down syndrome”* *“They told me [a healthcare professional] that he/she could be born with either the head very small or very big”*
By friends	*“Well, the first time I heard it from a friend and told me. Because in Brazil… because at that time we did not know, so she told me that some children were born with small heads. She did not tell me the name, just that they were born with small heads due to Zika, a lot of children were born like that. But I told her that we had to wait, he had to wait in name of Jesus Christ”*
Consequences on children’s health	
Perception of symptoms	*“I think that, as I did not have it [Zika] so strong, so deep, that there were some people with fever, diarrhea, and I did not have any of those symptoms. I just had small rash two days and that was all”*
Perception of severity	*“Because they said that that [Zika] happened if you were three months pregnant, that if three months or earlier, the baby could be born with problems, but as I had it when I was in my 5th month of pregnancy… Maybe because of that… [the baby is fine]”*
Stigmatization of the disease	*“And with Zika… imagine! Going with a deformed baby, that many people said that they were like little monsters. They told me that they were little monsters because they were born just with part of their face”*

## References

[1] Charlier C., Beaudoin M.C., Couderc T., Lortholary O., Lecuit M. (2017). Arboviruses and pregnancy: Maternal, fetal, and neonatal effects. Lancet Child Adolescent Health.

[2] Albuquerque M.D.F.P.M.D., Souza W.V.D., Araújo T.V.B., Braga M.C., Miranda Filho D.D.B., Ximenes R.A.D.A., de Melo Filho D.A., Brito C.A.A.D., Valongueiro S., Melo A.P.L.D. (2018). The microcephaly epidemic and Zika virus: Building knowledge in epidemiology. Epidemia de microcefalia e vírus Zika: A construção do conhecimento em epidemiologia. Cad. Saude Publica.

[3] Goncé A., Martínez M.J., Marbán-Castro E., Saco A., Soler A., Alvarez-Mora M.I., Peiro A., Gonzalo V., Hale G., Bhatnagar J. (2018). Spontaneous Abortion Associated with Zika Virus Infection and Persistent Viremia. Emerg. Infect. Dis..

[4] Shapiro-Mendoza C.K., Rice M.E., Galang R.R., Fulton A.C., VanMaldeghem K., Prado M.V., Ellis E., Anesi M.S., Simeone R.M., Petersen E.E. (2017). Zika Pregnancy and Infant Registries Working Group. Pregnancy Outcomes After Maternal Zika Virus Infection During Pregnancy—U.S. Territories, January 1, 2016–April 25, 2017. MMWR. Morb. Mortal. Wkly. Rep..

[5] van der Eijk A.A., van Genderen P.J., Verdijk R.M., Reusken C.B., Mögling R., van Kampen J.J., Widagdo W., Aron G.I., GeurtsvanKessel C.H., Pas S.D. (2016). Miscarriage Associated with Zika Virus Infection. N. Engl. J. Med..

[6] Musso D., Ko A.I., Baud D. (2019). Zika Virus Infection—After the Pandemic. N. Engl. J. Med..

[7] Walker C.L., Little M.T.E., Roby J.A., Armistead B., Gale M., Rajagopal L., Nelson B.R., Ehinger N., Mason B., Nayeri U. (2019). Zika virus and the nonmicrocephalic fetus: Why we should still worry. Am. J. Obstet. Gynecol..

[8] Einspieler C., Utsch F., Brasil P., Aizawa C.Y.P., Peyton C., Hasue R.H., Genovesi F.F., Damasceno L., Moreira M.E., Adachi K. (2019). Zika Working Group. Association of Infants Exposed to Prenatal Zika Virus Infection With Their Clinical, Neurologic, and Developmental Status Evaluated via the General Movement Assessment Tool. JAMA Netw. Open.

[9] Faiçal A.V., de Oliveira J.C., Oliveira J.V.V., de Almeida B.L., Agra I.A., Alcantara L.C.J., Acosta A.X., de Siqueira I.C. (2019). Neurodevelopmental delay in normocephalic children with in utero exposure to Zika virus. BMJ Paediatr. Open.

[10] Nielsen-Saines K., Brasil P., Kerin T., Vasconcelos Z., Gabaglia C.R., Damasceno L., Pone M., de Carvalho L.M.A., Pone S.M., Zin A.A. (2019). Delayed childhood neurodevelopment and neurosensory alterations in the second year of life in a prospective cohort of ZIKV-exposed children. Nat. Med..

[11] Broussard C.S., Shapiro-Mendoza C.K., Peacock G., Rasmussen S.A., Mai C.T., Petersen E.E., Galang R.R., Newsome K., Reynolds M.R., Gilboa S.M. (2018). Public Health Approach to Addressing the Needs of Children Affected by Congenital Zika Syndrome. Pediatrics.

[12] Kotzky K., Allen J.E., Robinson L.R., Satterfield-Nash A., Bertolli J., Smith C., Pereira I.O., Faria e Silva Santelli A.C., Peacock G. (2019). Depressive Symptoms and Care Demands Among Primary Caregivers of Young Children with Evidence of Congenital Zika Virus Infection in Brazil. J. Dev. Behav. Paediatr..

[13] Kuper H., Moreira M.E.L., de Araújo T.V.B., Valongueiro S., Fernandes S., Pinto M., Lyra T.M. (2019). The association of depression, anxiety, and stress with caring for a child with Congenital Zika Syndrome in Brazil; Results of a cross-sectional study. PLoS Negl. Trop. Dis..

[14] Lee M.H., Park C., Matthews A.K., Hsieh K. (2017). Differences in physical health, and health behaviors between family caregivers of children with and without disabilities. Disabil. Health J..

[15] Romero-Acosta K., Pérez D., Argumendos C., Barbosa J.L. (2019). Estudio de la interacción mamá-bebé prematuro a través de la escala Brazelton y algunas implicaciones sobre la salud mental de las Madres. Ocho Estudios de Salud Mental.

[16] Acosta K.R., Ruiz F. (2015). El trastorno de estrés postraumático en niños preescolares: Una revisión literaria. Katharsis.

[17] Klein P.S., Feldman R. (2007). Mothers’ and caregivers’ interactive and teaching behavior with toddlers. Early Child Dev. Care.

[18] Yamaoka Y., Tamiya N., Izumida N., Kawamura A., Takahashi H., Noguchi H. (2016). The relationship between raising a child with a disability and the mental health of mothers compared to raising a child without disability in japan. SSM-Popul. Health.

[19] Isa S.N.I., Ishak I., Ab Rahman A., Saat N.Z.M., Din N.C., Lubis S.H., Ismail M.F.M. (2016). Health and quality of life among the caregivers of children with disabilities: A review of literature. Asian J. Psychiatry.

[20] Masefield S.C., Prady S.L., Sheldon T.A., Small N., Jarvis S., Pickett K.E. (2020). The Caregiver Health Effects of Caring for Young Children with Developmental Disabilities: A Meta-analysis. Matern. Child Health J..

[21] Pilapil M., Coletti D.J., Rabey C., DeLaet D. (2017). Caring for the Caregiver: Supporting Families of Youth With Special Health Care Needs. Curr. Probl. Paediatr. Adolesc. Health Care.

[22] Edelstein H., Schippke J., Sheffe S., Kingsnorth S. (2017). Children with medical complexity: A scoping review of interventions to support caregiver stress. Child: Care, Health Dev..

[23] Teague S.J., Newman L.K., Tonge B.J., Gray K.M., MHYPeDD team (2018). Caregiver Mental Health, Parenting Practices, and Perceptions of Child Attachment in Children with Autism Spectrum Disorder. J. Autism Dev. Disord..

[24] dos Santos Oliveira S.J.G., de Melo E.S., Reinheimer D.M., Gurgel R.Q., Santos V.S., Martins-Filho P.R.S. (2016). Anxiety, depression, and quality of life in mothers of newborns with microcephaly and presumed congenital Zika virus infection. Arch. Women’s Ment. Health..

[25] Ebuenyi I.D., Bhuyan S.S., Bain L.E. (2018). Zika Virus infection and microcephaly: Anxiety burden for women. Pan Afr. Med. J..

[26] Freire I.M., Pone S.M., Ribeiro M.D.C., Aibe M.S., Pone M.V.D.S., Moreira M.E.L., Dupret L. (2018). Congenital Zika virus syndrome in infants: Repercussions for the promotion of families’ mental health. Síndrome congênita do Zika vírus em lactentes: Repercussões na promoção da saúde mental das famílias. Cad. Saude Publica.

[27] Vale P.R.L.F.D., Cerqueira S., Santos H.P., Black B.P., Carvalho E.S.D.S. (2019). Bad news: Families’ experiences and feelings surrounding the diagnosis of Zika-related microcephaly. Nurs. Inq..

[28] Williams N.A., Villachan-Lyra P., Marvin C., Chaves E., Hollist C., Hatton-Bowers H., Barbosa L.N.F. (2019). Anxiety and depression among caregivers of young children with Congenital Zika Syndrome in Brazil. Disabil. Rehabil..

[29] Arroyo K., Arrieta G., Mattar S., Romero K., Ramirez A. (2018). Exploración Del Neurodesarrollo a niños con y sin Microcefalia afectados por Virus de Zika en Sucre. Rev. Neuropsicol. Neuropsiquiatría Neurocienc..

[30] Romero-Acosta K. (2018). Esbozos sobre el método cualitativo y su aplicación a la investigación en salud mental. Rev. Epistemol. Psicol. Cienc. Soc..

[31] Spielberger C.D., Sydeman S.J., Owen A.E., Marsh B.J., Maruish M.E. (1999). Measuring anxiety and anger with the State-Trait Anxiety Inventory (STAI) and the State-Trait Anger Expression Inventory (STAXI). The Use of Psychological Testing for Treatment Planning and Outcomes Assessment.

[32] Lezama M.S.R. (2012). Propiedades psicométricas de la escala de Zung para síntomas depresivos en población adolescente escolarizada colombiana. Psychol. Av. Discip..

[33] Aragonès B.E., Masdéu M.R.M., Cando G.G., Coll B.G. (2001). Validez diagnóstica de la Self-Rating Depression Scale de Zung en pacientes de atención primaria. Actas Españolas de Psiquiatria.

[34] Valente S.M., Saunders J. (2005). Screening for depression and suicide: Self-report instruments that work. J. Psychosoc. Nurs. Ment. Health Serv..

[35] Villa G.I.C., Zuluaga A.C., Restrepo R.L.F. (2013). Propiedades psicométricas del Cuestionario de Salud General de Goldberg GHQ-12 en una institución hospitalaria de la ciudad de Medellín. Avances en Psicología Latinoamericana.

[36] Gottfried A.W. (1985). Measures of Socioeconomic Status in Child Development Research: Data and Recommendations. Merrill-Palmer Q..

[37] DeSilva M., Munoz F.M., Sell E., Marshall H., Kawai A.T., Kachikis A., Heath P., Klein N.P., Oleske J.M., Jehan F. (2017). Congenital microcephaly: Case definition & guidelines for data collection, analysis, and presentation of safety data after maternal immunisation. Vaccine.

[38] Guo F., Norton A.R., Fuchs E.L., Hirth J.M., Garcia-Blanco M.A., Berenson A.B. (2017). Provider-patient communication about Zika during prenatal visits. Prev. Med. Rep..

[39] Pooransingh S., Parasram R., Nandram N., Bhagwandeen B., Dialsingh I. (2018). Zika virus disease—knowledge, attitudes and practices among pregnant women—implications for public health practice. Public Health.

[40] Matos T.D.D.S., Meneguin S., Ferreira M.D.L.D.S., Miot H.A. (2017). Quality of life and religious-spiritual coping in palliative cancer care patients. Rev. Lat. Am. Enferm..

[41] Ekas N.V., Whitman T.L., Shivers C. (2009). Religiosity, spirituality, and socioemotional functioning in mothers of children with autism spectrum disorder. J. Autism Dev. Disord..

